# Perceived Quality-of-Life Importance Among Saudi Gynecologic Cancer Survivors: Latent Class Analysis

**DOI:** 10.3390/curroncol32100557

**Published:** 2025-10-04

**Authors:** Wedad M. Almutairi, Fatmah Alsharif, Ahlam Al-Zahrani, Noura Bin Afeef, Alkhnsa Alkeai, Haneen Alfakeeh, Arwa Alzahrani, Nouran Essam Katooa, Fathia Khamis Kassem, Wafa A. Faheem

**Affiliations:** 1 Maternity and Child Health Department, Faculty of Nursing, King Abdulaziz University, Jeddah 21589, Saudi Arabia; aealzahrani@kau.edu.sa (A.A.-Z.); nbinafeef@stu.kau.edu.sa (N.B.A.); aalkeai0001@stu.kau.edu.sa (A.A.); halfakeeh0003@stu.kau.edu.sa (H.A.); aalzahrani3124@stu.kau.edu.sa (A.A.); nkuttouaha@kau.edu.sa (N.E.K.); wfaheem@kau.edu.sa (W.A.F.); 2 Medical Surgical Nursing Department, Faculty of Nursing, King Abdulaziz University, Jeddah 21589, Saudi Arabia; falsharif@kau.edu.sa; 3 Public Health Department, Faculty of Nursing, King Abdulaziz University, Jeddah 21589, Saudi Arabia; fkibrahim@kau.edu.sa; 4 Community Health Nursing Department, Alexandria University, Alexandria 21526, Egypt

**Keywords:** gynecologic cancer survivors, Quality of Sexual Life, latent class analysis, sexual health, decision tree modeling

## Abstract

Quality-of-life (QoL) needs among gynecologic cancer survivors are multifaceted and culturally mediated, yet limited research has examined how survivors in the Middle East prioritize key domains such as sexual function, emotional well-being, and relational quality. This study aimed to identify subgroups of survivors based on the perceived importance of these domains and to explore demographic and clinical predictors of subgroups within the Saudi Arabian context. We conducted a cross-sectional, survey-based study among 129 women with a history of breast or cervical cancer attending a tertiary oncology center in Jeddah, Saudi Arabia. Participants rated the importance of sexual, emotional, and relational QoL domains using a 4-point Likert scale. Latent class analysis (LCA) was used to segment survivors based on their perceived domain importance. Differences in demographic and clinical characteristics across classes were assessed using chi-square tests. A decision tree classifier was employed. Three latent classes emerged: Class 0 (48.8%) prioritized all domains highly; Class 1 (17.8%) reported low importance across domains; and Class 2 (33.3%) emphasized emotional and relational domains while downplaying sexual function. Class group was significantly associated with age (*p* = 0.001), education (*p* = 0.04), nationality (*p* = 0.03), and number of children (*p* < 0.001). Decision tree analysis identified number of children, age, and marital status as the strongest predictors of high-importance class group. Gynecologic cancer survivors in Saudi Arabia hold diverse priorities regarding QoL domains, primarily shaped by sociocultural context than clinical variables. Tailored survivorship interventions that reflect survivors’ lived values, particularly in relation to age, family structure, and cultural norms, are critical for person-centered oncology care in the region.

## Simple Summary

This study looked at what matters most to women in Saudi Arabia who have survived gynecologic cancers such as breast or cervical cancer. The researchers asked 129 women to rate how important different aspects of life are to them after treatment—especially their emotional well-being, relationships, and sexual health. The women were grouped based on their answers. Some valued all aspects highly, while others focused more on emotional or relationship needs and less on sexual health. These priorities were influenced more by personal and cultural factors—like age, family size, and education—than by their medical history. Understanding what survivors care about most can help healthcare providers design better, more personalized support services. The findings can guide future research and healthcare policies to better match women’s real-life needs during cancer recovery in culturally sensitive ways.

## 1. Introduction

Gynecologic cancers including malignancies of the cervix, uterus, ovaries, and vulva pose a considerable health challenge to women worldwide. In Saudi Arabia, cervical and breast cancers remain among the most frequently diagnosed malignancies in women, with a growing number of survivors due to improvements in early detection and access to more effective treatment options [1,2]. However, while clinical outcomes have steadily advanced, broader quality-of-life (QoL) concerns, particularly those related to sexual function, emotional well-being, and interpersonal relationships, have not received equivalent attention. These concerns are especially under-addressed in culturally conservative settings, where open dialog about intimacy, mental health, and relational dynamics is often constrained by social norms [3,4]. Cancer treatments such as surgery, chemotherapy, radiation, and hormone therapy often affect survivors in deeply personal ways. Many women report changes such as reduced sexual desire, vaginal dryness, discomfort during intimacy, and a shifting sense of body image [3,4]. These physical effects frequently intersect with emotional outcomes, including anxiety, depression, and a sense of social withdrawal factors that, when left unaddressed, can hinder recovery and diminish survivors’ overall well-being [5,6].

In Saudi society, these challenges are often compounded by cultural expectations around modesty, privacy, and gender roles. Topics like sexual health and emotional vulnerability are often considered private or taboo, making it difficult for women to voice their needs or access support [7]. In clinical practice, such discussions may be avoided altogether due to time constraints or discomfort among healthcare providers [8]. Consequently, little is known about how survivors themselves rank the importance of different QoL domains, and even less is understood about how these priorities differ across women based on their age, marital status, or treatment experience. To bridge this knowledge gap and move toward more person-centered care, it is critical to identify what survivors value most during recovery. Not all women experience survivorship in the same way, and assuming uniformity in their needs may lead to missed opportunities for meaningful support. Latent class analysis (LCA) provides a powerful analytic framework for addressing this issue. Unlike traditional methods that focus on group averages, LCA allows researchers to uncover hidden subgroups within a population who share similar response patterns—offering a more nuanced view of survivor experiences and priorities [9].

This study applies LCA to a cohort of Saudi women who have completed treatment for gynecologic cancers. Our aims were twofold: first, to identify unobserved subgroups based on how participants rated the importance of sexual, emotional, and relational aspects of their quality-of-life; and second, to describe these subgroups in relation to their demographic and clinical characteristics. Furthermore, we explored the most influential predictors of belonging to the high-importance class. This analysis not only helps in identifying who prioritizes sexual, emotional, and relational well-being the most, but also sheds light on why certain survivor profiles exhibit stronger quality-of-life expectations in these domains. By revealing the diversity of survivors’ values, this research may help clinicians and policymakers design support strategies that are not only evidence-based but also culturally and personally relevant to women in Saudi Arabia.

In Saudi Arabia, national cancer registry reports and prior epidemiological studies confirm that breast and cervical cancers remain among the most prevalent malignancies in women, accounting for a substantial proportion of the cancer burden [1,2]. While some Saudi and Middle Eastern studies have explored aspects of survivorship—such as general health-related QoL, sexual well-being, and psychosocial challenges—these have primarily reported aggregated group averages and have rarely examined how survivors prioritize different QoL domains in culturally conservative settings [3,4,5,6,7,8]. Importantly, no published Saudi studies have applied latent class analysis (LCA) or similar person-centered statistical approaches to segment survivors based on self-reported importance of QoL domains, despite growing international recognition of the value of such methods in tailoring survivorship care [9]. This lack of segmentation data limits the ability of healthcare providers to design targeted, culturally relevant interventions that address the diverse needs of survivors. The present study addresses this gap by applying LCA to a cohort of Saudi breast and cervical cancer survivors to identify subgroups defined by their QoL priorities and to explore demographic and clinical predictors of subgroup membership, thereby contributing novel, context-specific evidence to the regional survivorship literature.

## 2. Materials and Methods

**Study Design and Setting:** This cross-sectional, survey-based study aimed to identify unobserved subgroups of gynecologic cancer survivors based on the perceived importance they place on sexual function and satisfaction, psychological well-being, and social relationship quality. This was a single-center study conducted at a tertiary oncology referral hospital, King Abdulaziz Medical City in Jeddah, Saudi Arabia, between May and July 2021 [10].

**Participants and Eligibility Criteria:** Women aged 18 years or older with a confirmed diagnosis of breast or cervical cancer who had completed or were undergoing primary treatment (surgery, chemotherapy, or radiation) were eligible. Participants were required to be fluent sin reading and speaking Arabic to complete the questionnaire independently. Women with active cancer recurrence were excluded. Time since diagnosis was recorded and categorized as <1 year, 1–3 years, or >3 years to capture differences across survivorship stages. Fluency in reading and speaking Arabic was confirmed through a screening question to ensure participants could complete the questionnaire independently without assistance.

Time since diagnosis was recorded for all participants and categorized as less than 1 year, 1–3 years, or more than 3 years, serving as a proxy for survivorship stage.

**Recruitment and Data Collection Procedure:** Participants were recruited during routine oncology follow-up visits at King Abdulaziz Medical City, Jeddah, and through targeted social media advertisements restricted to current or former patients of the same center. After providing informed electronic consent, participants completed the survey via a secure, web-based platform in a private setting, either on-site or remotely.

Participants recruited via social media were limited to current or former patients of the oncology clinics at King Abdulaziz Medical City, Jeddah, and eligibility was confirmed through self-reported medical history, including diagnosis and treatment status at the center.

### Survey Instrument and Domain Importance Ratings

The survey instrument consisted of four structured sections. The first three sections assessed survivors’ satisfaction and functioning across three core quality-of-life (QoL) domains:

1. Sexual Function and Satisfaction (8 items);
2. Psychological and Emotional Well-Being (3 items);
3. Social and Relationship Quality (3 items).

These items were adapted from two validated instruments: the Satisfaction Survey [11,12], developed to assess sexual satisfaction and function in gender-diverse populations post-vaginoplasty, and the Quality of Sexual Life Questionnaire for breast cancer survivors in Mainland China [4]. Both original instruments are designed to assess current functional status and satisfaction within their respective domains. A fourth section was developed specifically for this study to assess the perceived importance of each QoL domain. Participants were asked to rate how important each domain was to them using a 4-point Likert scale (1 = not important to 4 = extremely important). This importance-rating section was not part of the original validated instruments [4,11,12], which focused exclusively on assessing satisfaction and functional outcomes. The addition of this fourth section allowed for the assessment of value attribution, enabling segmentation based on survivors’ self-defined QoL priorities. This adaptation is consistent with emerging recommendations in patient-reported outcome (PRO) methodology, which emphasize capturing not only patient experiences but also what patients personally value during survivorship care [13]. By integrating this importance-rating component, the instrument enabled latent class analysis (LCA) to segment participants based on their individually prioritized QoL domains, supporting a culturally sensitive and patient-centered analytic approach.

**Translation, Content Validation, and Reliability:** The survey was translated into Arabic using WHO-recommended forward and backward translation procedures [14,15]. It was reviewed by bilingual experts and piloted with 20 Saudi women to ensure cultural relevance and clarity. Content modifications were made to enhance readability. While adapted from validated instruments and translated following WHO guidelines, the combined instrument used in this study has not undergone psychometric validation. But, The final version demonstrated high internal consistency (Cronbach’s alpha = 0.89), supporting its reliability for group-level analysis [16].

**Sample Size and Sampling Technique:** Given the lack of prior segmentation studies in Saudi cancer survivors, sample size adequacy was determined following established recommendations for latent class analysis to ensure sufficient power for meaningful subgroup identification, despite the absence of prior data on how such segments might emerge in this population.

Sample size was calculated based on recommendations for latent class analysis and multivariable modeling, with a target of at least 100 participants to allow for meaningful segmentation and exploratory modeling [17]. A total of 129 women completed the survey (response rate = 90%), exceeding the minimum required. Participants were recruited via convenience sampling during outpatient oncology visits [18].

**Data Collection and Ethical Considerations:** The study was approved by the Nursing Research Ethical Committee at the Faculty of Nursing, King Abdulaziz University (Ref No 1F.21). Data were collected anonymously through the secure online platform, with no researcher involvement in data entry. All clinical and demographic information was self-reported by participants to maintain privacy and standardization.

**Statistical Analysis:** Latent class analysis (LCA) was used to identify unobserved subgroups of gynecologic cancer survivors based on their perceived importance of three domains of quality-of-life: sexual function and satisfaction, psychological and emotional well-being, and social and relationship quality. These domains were measured using a 4-point Likert scale (1 = not important to 4 = extremely important). The LCA approach follows best practices for population segmentation using patient-reported outcomes and has been widely adopted in psycho-oncology and survivorship care research [19].

We applied a Gaussian Mixture Model (GMM) framework, suitable for continuous or ordinal measures that approximate normal distributions. Models were estimated iteratively for 1 to 6 latent classes. The optimal number of classes was determined by comparing the Bayesian Information Criterion (BIC) and Akaike Information Criterion (AIC) across models. BIC was used as the primary selection criterion, consistent with guidance from prior work emphasizing parsimony and model fit balance in latent class modeling [20].

To assess classification accuracy, we computed the posterior class probabilities for each participant and assigned them to the class with the highest likelihood. Model separation and assignment certainty were evaluated using the Silhouette Score and entropy. The Silhouette Score quantifies how well-separated individuals are across clusters, with values >0.5 considered good separation. Entropy captures classification uncertainty and approaches zero when probabilities are near deterministic (i.e., close to 0 or 1) [21,22].

After determining the final three-class solution, we assessed the distribution of key demographic and clinical characteristics across latent classes using chi-square tests or Fisher’s exact test when expected frequencies were below 5. This allowed us to evaluate whether class group was significantly associated with factors such as age, marital status, education, number of children, nationality, and cancer-related history [22].

## 3. Results

**Figure 1:**
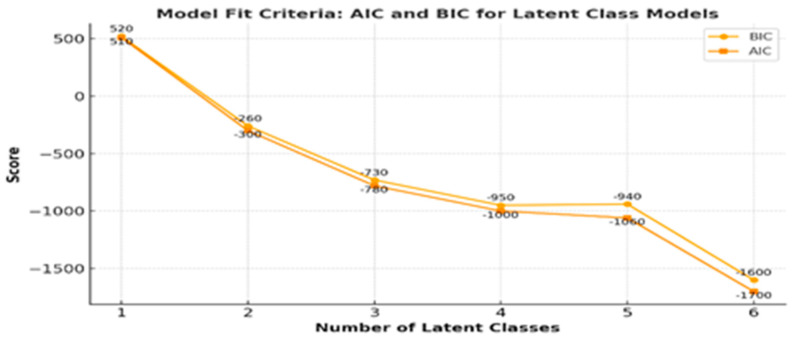
Model fit criteria.

Latent class analysis (LCA) was conducted to identify unobserved subgroups of Saudi gynecologic cancer survivors based on the perceived importance of three quality-of-life domains: sexual function and satisfaction, psychological and emotional well-being, and social and relationship quality. Model fit was evaluated across latent class solutions ranging from 1 to 6 classes using log-likelihood, Akaike Information Criterion (AIC), Bayesian Information Criterion (BIC), entropy, and posterior class probabilities (Table 1). Both AIC and BIC values improved markedly from the 1-class model (AIC = 510; BIC = 520) to the 3-class model (AIC = −780; BIC = −730), indicating enhanced fit with additional classes. Although the 6-class model yielded the lowest AIC (−1700) and BIC (−1600), it introduced multiple small and sparsely populated classes (e.g., class probabilities = 0.016, 0.054). Entropy, which quantifies classification certainty, increased across models and reached 0.84 in the 3-class solution, suggesting good separation between classes. While entropy continued to rise in more complex models (e.g., 0.98 for 6 classes), the 3-class model offered a well-balanced distribution of survivors (class probabilities = 0.17, 0.48, 0.33) and aligned conceptually with the study objectives capturing distinct survivor subgroups based on their valuation or perceived importance of sexual, emotional, and relational quality-of-life domains (Table 1 and Figure 1).

### 3.1. Interpretation of Response Probabilities by Latent Class Analysis

**Figure 2:**
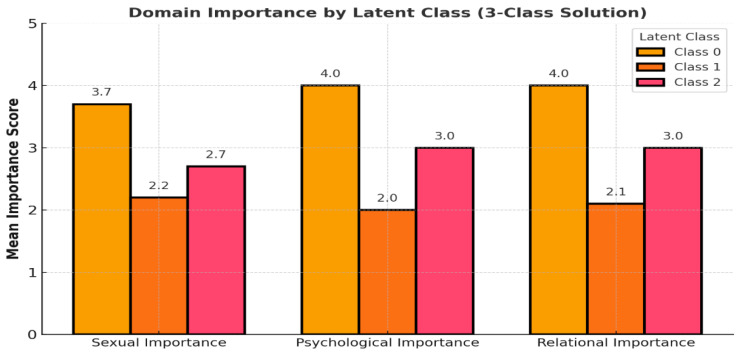
Response probabilities by latent class solution. The figure displays the average perceived importance of three quality-of-life domains—sexual, psychological, and relational—across the three latent classes identified via latent class analysis. Class 0 rated all domains as highly important, with mean scores approaching the maximum value of 4, indicating uniform prioritization. Class 1 assigned the lowest importance to all domains, suggesting diminished emphasis on psychosocial and sexual well-being. Class 2 prioritized psychological and relational domains while assigning lower importance to sexual well-being. These domain-level response patterns validate the latent class structure and underscore the heterogeneity in survivors’ quality-of-life priorities.

The 3-class latent class solution revealed distinct patterns of prioritization across quality-of-life (QoL) domains—sexual, emotional, and relational. Class 0 (48.8%), the largest subgroup, exhibited uniformly high importance scores across all domains, with mean values ranging from 3.7 to 4.0. This class reflects a group of survivors who place consistently strong value on all aspects of post-cancer quality-of-life, suggesting a comprehensive orientation toward recovery and well-being. Class 1 (17.8%) showed moderate to low importance scores, particularly in the sexual function and relational quality domains, where scores averaged around 2.0–2.2. This group appears to reflect survivors who may be more reserved or disengaged from sexual or interpersonal QoL dimensions potentially due to cultural, psychological, or treatment-related factors that suppress such needs or expectations. Class 2 (33.3%) demonstrated asymmetrical prioritization, assigning highest importance to relational (mean = 3) while valuing sexual and psychological well-being domains more moderately (means~2.7–3.0). This pattern suggests a subgroup that prioritizes emotional recovery as central to their survivorship experience, possibly indicating residual psychological distress or a preference for internal coping over interpersonal or sexual re-engagement (Figure 2).

### 3.2. Sociodemographic and Clinical Profiles by Latent Class Group

Latent class analysis identified three distinct subgroups of Saudi gynecologic cancer survivors based on their quality-of-life importance ratings across sexual, emotional, and relational domains. Class 0 (48.8%) represented survivors who assigned uniformly high importance across all domains; Class 1 (17.8%) reflected low importance ratings; and Class 2 (33.3%) prioritized emotional and relational well-being while placing lower importance on sexual functioning. The distribution of demographic and clinical characteristics across classes (Table 2 and Figure 2) revealed significant and clinically meaningful differences. Age was strongly associated with class group (*p* = 0.001), with younger women more frequently classified into Class 0, suggesting evolving sexual priorities during early survivorship. Conversely, older survivors were more often in Class 2, potentially reflecting asymmetrical prioritization, assigning highest importance to social relationship with silence around sexual health. Educational level differed significantly across classes (*p* = 0.04), with Class 0 comprising a higher proportion of women with university-level education. Similarly, nationality was associated with latent class (*p* = 0.03): Class 0 was composed primarily of Saudi nationals (96.8%), while Class 2 included a greater share of non-Saudis (18.6%), possibly reflecting disparities in healthcare access or cultural differences in expressing or valuing quality-of-life dimensions. Number of children showed the strongest association (*p* < 0.001), with survivors in Class 0 more likely to have either no children or a moderate number [1,2,3], while those in Class 2 frequently had larger families [4,5,6,7,8,9,10]. This pattern underscores how familial roles may either constrain or elevate the perceived importance of relational and emotional dimensions of quality-of-life. The number of children was significantly associated with latent class membership; however, the present study did not investigate the underlying reasons for this association.

Additional significant differences were observed for age at diagnosis (*p* = 0.03), and time since diagnosis (*p* = 0.05). Women diagnosed at a younger age or more recently were more often found in Class 1, and 2, reflecting a survivor profile that may prioritize emotional coping and relationship stability over sexual function in the early phases of recovery.

### 3.3. Predictors of High-Importance Class Group (Non-Parametric Analysis)

To identify the strongest predictors of belonging to the high-importance subgroup (Class 0), we conducted a non-parametric decision tree classification. The model identified Number of Kids (18.4%) as the most influential variable. This reinforces the role of family structure in shaping survivors’ quality-of-life priorities potentially reflecting either the burden of caregiving or the emotional centrality of familial relationships.

Age (17.8%) and Marital Status (16.9%) also contributed meaningfully. Younger survivors may be more concerned with intimacy and sexual health, while married individuals may place greater emphasis on relational dynamics and mutual emotional support. Time since diagnosis (14.2%) and age at diagnosis (12.9%) were also relevant, indicating that both disease chronology and life stage at onset influence survivors’ quality-of-life frameworks.

Notably, occupational status and educational level, while influential in the chi-square analysis, showed relatively lower importance in the tree-based model, suggesting their effects may be mediated or moderated by other more proximal factors like age, family size, or diagnosis timing.

### 3.4. Summary

**Figure 3:**
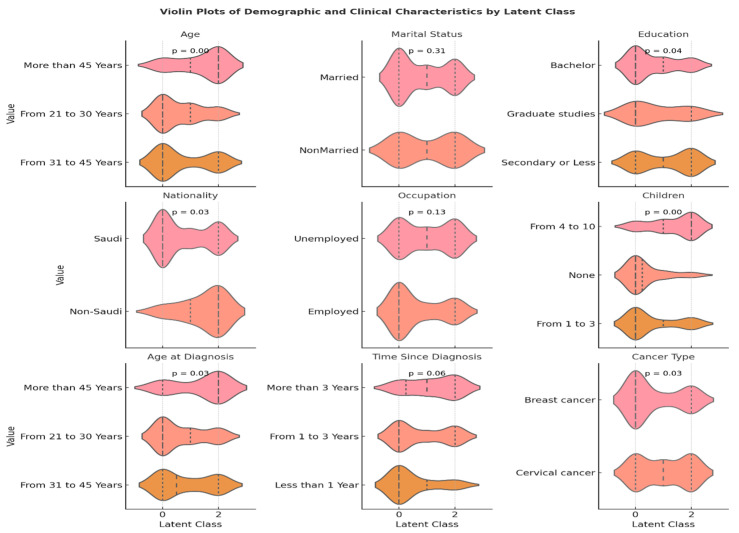
Violin plot of the distribution of demographic and clinical characteristics across latent classes of quality-of-life importance among Saudi gynecologic cancer survivors.

**Figure 4:**
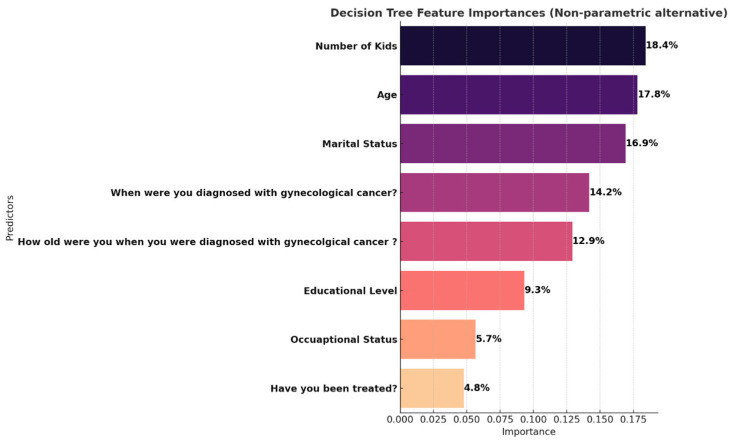
Decision tree feature.

Together, these results validate the three-class latent model not only in statistical terms (entropy = 0.68, BIC = 827.29) but also in clinical relevance. Each class represents a unique survivor profile that can inform targeted psychosocial interventions. Programs that consider age, family context, marital status, and survivorship stage may be most effective in aligning support services with survivors’ lived experiences and quality-of-life priorities (Figure 3 and Figure 4).

## 4. Discussion

This study sought to identify unobserved subgroups of Saudi gynecologic cancer survivors based on the perceived importance of three core domains of quality-of-life: sexual function and satisfaction, psychological and emotional well-being, and social and relationship quality. By applying latent class analysis to a sample of Saudi gynecologic cancer survivors and further examining the sociodemographic and clinical correlates of class group using decision tree modeling, we gained novel insights into how survivors in Saudi Arabia differentially prioritize various aspects of their well-being. These findings underscore the importance of incorporating value-based patient-reported outcomes in survivorship care models, particularly within culturally specific contexts. Our results echo similar findings from latent class analyses conducted among young breast cancer patients in China [23], where five distinct sexual health subgroups were identified. Although our study focuses on QoL importance rather than functional impairments, the structure of the latent classes reflects underlying psychosocial complexities consistent with earlier international studies. Yuan et al. noted variations in sexual activity and satisfaction as distinguishing features, while our study captured perceived prioritization of domains. This distinction is essential in Middle Eastern including Saudi contexts, where discussing sexual concerns remains culturally sensitive and may not be openly disclosed or prioritized unless explicitly asked [8].

Our results revealed a three-class solution that demonstrated both statistical fit and clinical interpretability. Class 0, comprising nearly half the sample (48.8%), was characterized by uniformly high importance ratings across all domains. This group reflects survivors who remain highly attuned to their psychosocial and sexual health needs post-treatment, and likely represent a subgroup motivated to maintain or restore a sense of comprehensive well-being. Notably, women in this class were more likely to have higher educational attainment, be of Saudi nationality, and have fewer children compared to the other classes. Similar patterns have been noted in previous studies from Western and East Asian populations, where younger and educated women demonstrate greater engagement with psychosocial survivorship services [24,25]. This subgroup likely benefits from comprehensive, integrative support programs that recognize the intersection of sexual, emotional, and relational health.

In contrast, Class 1 (17.8%) included survivors who rated all three domains as low in importance. This subgroup was more likely to include older women, individuals with lower educational attainment, and non-Saudi nationals. These findings raise concerns regarding access, empowerment, and cultural comfort in discussing sensitive topics such as sexual health. Previous studies in conservative settings have shown similar underreporting of sexual and emotional concerns [26]. The high prevalence of unmet psychosocial needs in this subgroup suggests a need for structured clinician-initiated discussions and increased access to psychoeducational interventions. The low prioritization of QoL domains in this class may also be symptomatic of deeper psychosocial distress or disengagement from survivorship identity, both of which merit clinical attention. Class 2’s asymmetrical prioritization pattern moderate emphasis on emotional and relational domains and lower prioritization of sexual function mirrors findings in other LCA studies among survivors undergoing endocrine therapy. For example, studies have demonstrated that patients often delay sexual reintegration during the early stages of survivorship, focusing initially on emotional recovery and relational cohesion [27,28]. In our study, this class was more likely to include women diagnosed recently or at a younger age, supporting the notion that QoL priorities evolve over the survivorship trajectory. These survivors may benefit from phased intervention strategies, beginning with emotional support and gradually integrating sexual health counseling.

Across all three classes, marital status, employment, and cancer type did not differ significantly, though trends suggest that being married or employed may offer a supportive structure that facilitates prioritization of certain quality-of-life domains. The lack of strong associations between clinical variables (e.g., cancer type, treatment status) and domain importance underscores that survivorship experiences are shaped more by sociocultural and demographic contexts than by disease characteristics alone.

To complement the LCA findings, we used a decision tree classifier to identify which variables best predict group in the high-importance group (Class 0). The most influential predictor was number of children, followed by age and marital status. Women with fewer children were more likely to assign high importance across all domains, possibly due to reduced caregiving demands or greater ability to invest in personal well-being. The number of children emerged as a significant demographic factor associated with QoL priority classifications in our analysis. While this is an important observation, the present study did not examine why this association exists, and any interpretation of potential mechanisms would require dedicated, hypothesis-driven research in future studies.

Age and marital status also emerged as meaningful predictors, reinforcing that life stage and relational context significantly shape survivors’ perspectives on recovery. These findings have direct implications for clinical practice: survivorship care plans should be individualized not only by diagnosis and treatment history but also by personal and relational factors that influence patient priorities. Importantly, this study is among the first to apply latent class modeling to perceived quality-of-life importance among cancer survivors in Saudi Arabia. By situating the findings within the Saudi cultural context, our work addresses a critical gap in global survivorship research, which remains largely Western-centric. The results highlight the need to move beyond a one-size-fits-all approach to survivorship care. Programs that emphasize shared decision-making, personalized goal-setting, and culturally sensitive support structures are likely to yield better alignment with survivors’ lived experiences and values.

### Limitations

Several limitations warrant consideration. First, the sample was drawn from a single tertiary care center in Jeddah and may not reflect the full diversity of survivors across Saudi Arabia, particularly those in rural or underserved areas. Second, reliance on self-reported data introduces the possibility of social desirability bias, especially around topics considered sensitive. Third, while the decision tree model provided insights into predictors of class group, it does not offer causal inferences and should be interpreted as exploratory. Additionally, although the survey items were adapted from validated instruments and underwent rigorous translation and pilot testing, the combined instrument has not been fully psychometrically validated, which may limit the generalizability of the findings.

It is important to note that survivorship needs may evolve over time. The present study included participants at varying stages since diagnosis, and differences in time from treatment completion could influence how quality-of-life domains are prioritized. Future longitudinal studies are needed to capture these changes and inform stage-specific survivorship care. This also study did not collect detailed information on treatment type or clinical stage of disease, both of which may influence survivors’ quality-of-life priorities; this should be considered a limitation of our findings. As this is an observational study, the reasons underlying the observed associations were not explored in depth, and interpretations should be viewed as exploratory.

## 5. Conclusions

This study provides novel evidence that gynecologic cancer survivors in Saudi Arabia hold heterogeneous views on what aspects of quality-of-life matter most during survivorship. Through latent class analysis, we identified three distinct subgroups: those prioritizing all domains highly, those deprioritizing them, and those selectively emphasizing emotional and relational well-being. These classes were shaped not by clinical characteristics alone, but by sociodemographic and cultural factors, including age, education, nationality, and family structure. Non-parametric decision tree modeling confirmed that number of children, age, and marital status were key predictors of high-importance class group. These findings underscore the need for culturally contextualized, patient-prioritized survivorship care pathways in Saudi Arabia. Tailored interventions that align with survivors’ values and lived realities are essential for delivering equitable, person-centered cancer care across diverse cultural settings.

## Figures and Tables

**Table 1:** Model fit regarding the indices of latent class analysis models.

Classes	Log Likelihood	AIC ^a^	BIC ^b^	Entropy	Class Probabilities
1 class	−237.54	510	520	---	---
2 class	183.63	−300	−260	0.81	0.512, 0.488
3 class	439.63	−780	−730	0.84	0.178, 0.488, 0.333
4 class	464.59	−1000	−950	0.91	0.155, 0.488, 0.333, 0.023
5 class	606.77	−1060	−940	0.96	0.155, 0.488, 0.008, 0.016, 0.333
6 class	617.45	−1700	−1600	0.98	0.155, 0.442, 0.140, 0.016, 0.194, 0.054

^a^ AIC: Akaike information criterion. ^b^ BIC: Bayesian information criterion.

**Table 2:** Distribution of demographic and clinical characteristics across latent classes of quality-of-life importance among Saudi gynecologic cancer survivors.

Variable	Category	Class 0 ^a^ (n, %)	Class 1 ^b^ (n, %)	Class 2 ^c^ (n, %)	*p*-Value ^d^
**Total**	-----------	63 (48.83%)	23 (17.8%)	43 (33.3%)	
**Age**	From 21 to 30 Years	23 (36.5%)	12 (52.2%)	8 (18.6%)	0.001
	From 31 to 45 Years	34 (54.0%)	6 (26.1%)	19 (44.2%)	
	More than 45 Years	6 (9.5%)	5 (21.7%)	16 (37.2%)	
**Marital Status**	Married	53 (84.1%)	21 (91.3%)	33 (76.7%)	0.31
	Nonmarried	10 (15.9%)	2 (8.7%)	10 (23.3%)	
**Educational Level**	Bachelor	35 (55.6%)	14 (60.9%)	13 (30.2%)	0.04
	Graduate studies	7 (11.1%)	2 (8.7%)	4 (9.3%)	
	Secondary or Less	21 (33.3%)	7 (30.4%)	26 (60.5%)	
**Nationality**	Non-Saudi	2 (3.2%)	3 (13.0%)	8 (18.6%)	0.03
	Saudi	61 (96.8%)	20 (87.0%)	35 (81.4%)	
**Occupational Status**	Employed	33 (52.4%)	8 (34.8%)	15 (34.9%)	0.13
	Unemployed	30 (47.6%)	15 (65.2%)	28 (65.1%)	
**Number of kids**	From 1 to 3	38 (60.3%)	7 (30.4%)	14 (32.6%)	0.0001
	From 4 to 10	10 (15.9%)	13 (56.5%)	27 (62.8%)	
	None	15 (23.8%)	3 (13.0%)	2 (4.7%)	
**Age when you first diagnosis**	From 21 to 30 Years	33 (52.4%)	13 (56.5%)	14 (32.6%)	0.03
	From 31 to 45 Years	24 (38.1%)	8 (34.8%)	16 (37.2%)	
	More than 45 Years	6 (9.5%)	2 (8.7%)	13 (30.2%)	
**When were you diagnosed with gynecological cancer?**	From 1 to 3 Years	40 (63.5%)	13 (56.5%)	26 (60.5%)	0.05
	Less than 1 Year	16 (25.4%)	3 (13.0%)	4 (9.3%)	
	More than 3 Years	7 (11.1%)	7 (30.4%)	12 (27.9%)	
**Type of diagnosed cancer**	Breast cancer	38 (60.3%)	7 (30.4%)	18 (41.9%)	0.03
	Cervical cancer	25 (39.7%)	16 (69.6%)	25 (58.1%)	

^a^ Class 0 showed high importance ratings across all domains (sexual, emotional, and relational), ^b^ Class 1 showed low importance ratings, and ^c^ Class 2 prioritized emotional and relational well-being while rating sexual importance lower. ^d^ chi-square test or fisher exact test for frequency lower than 5.

## Data Availability

All data generated or analyzed during the current study are fully available within this manuscript. The dataset supporting the conclusions, including the descriptive statistics and latent class analysis outputs, has been presented in the tables and figures provided. No additional external repositories or supplementary datasets were created or required, as the study findings are entirely based on the data presented herein.

## Abbreviations

The following abbreviations are used in this manuscript:

DOAJ	Directory of open access journals
TLA	Three-letter acronym
LD	Linear dichroism
